# Glycerol photoelectrochemical oxidation reaction at carbon nitrides/BiVO_4_ materials

**DOI:** 10.3762/bjnano.17.57

**Published:** 2026-06-17

**Authors:** Charles Garcia da Cunha, Isabelle M D Gonzaga, Cristian Hessel, Izadora F Reis, Ivo F Teixeira, Lucia H Mascaro, Elton Sitta

**Affiliations:** 1 Federal University of São Carlos, São Carlos, SP, Brazil

**Keywords:** biomass valorization, bismuth vanadate, glycerol valorization, heterojunctions, photoelectrochemistry

## Abstract

The H_2_ evolution from water electrolysis can be coupled with co-generation of other added-value products through biomass oxidation. In this study, we investigate the photoelectrochemical oxidation of glycerol using visible-light-responsive carbon nitride /bismuth vanadate (CN/BiVO_4_) heterojunction photoelectrocatalysts. Different CN materials were explored, including polymeric carbon nitride (PCN), crystalline poly(heptazine imides) (PHI-Cat, in which Cat = Na, K, or Cs), and poly(triazine imide) (PTI-Li). The CN materials were spin-coated onto fluorine-doped tin oxide (FTO) substrates, followed by the Bi electrodeposition step and conversion to BiVO_4_ in the presence of vanadyl acetylacetonate at 500 °C for 2 h. The CN/BiVO_4_ heterojunctions presented bandgap energy values, *E*_g_, similar to pure BiVO_4_. X-ray diffraction analysis also revealed that the BiVO_4_ phase was not altered by the presence of the CN. However, scanning electron microscopy analysis coupled to energy-dispersive X-ray spectroscopy (SEM-EDS) revealed regions rich in Bi and V, and others rich in C and N, suggesting the formation of heterojunctions. Photoelectrochemical studies demonstrated that BiVO_4_ is active for both water and glycerol (1.0 mol·L^−1^) oxidation, with a fourfold increase in photocurrent at 1.23 V vs reversible hydrogen electrode (RHE) upon glycerol addition. Clearly, the type of nitride employed in the heterojunctions influences the activity of the material for glycerol oxidation, with the photocurrent at 1.23 V vs RHE following the order: PCN/BiVO_4_ > BiVO_4_ ≈ PHI-Cs/BiVO_4_ > PHI-K/BiVO_4_ ≈ PHI-Na/BiVO_4_ > PTI(Li)/BiVO_4_. The SEM-EDS analysis after electrochemical tests revealed that the presence of crystalline CNs induces the segregation of vanadium oxides, contributing to a decrease in activity. On the other hand, the superior performance of PCN/BiVO_4_ is attributed to a greater thermal stability of PCN during BiVO_4_ synthesis, as indicated by thermogravimetric analysis. These findings highlight the dual importance of electronic compatibility and thermal resilience of CN materials in designing efficient heterojunction photoanodes for biomass-assisted hydrogen production.

## Introduction

Hydrogen production based on sustainable and renewable energy sources (the so-called green-H_2_) is a promising alternative for energy storage, with potential applications in fuel cells and the chemical industry [1]. Among the various methods of producing green-H_2_ [2], water photoelectrolysis [3] allows the direct use of solar energy, decreasing reliance on fossil fuels, and having potential for large-scale hydrogen production [4]. On the other hand, the challenges related to the sluggish kinetics of the water oxidation reaction, as well as the material stability at the anodes, must be overcome for this technology to be successfully transferred to end users [5].

To address these issues, several efforts have been made to develop efficient catalysts to improve the kinetics of the water oxidation reaction [6]. These strategies include the synthesis of new photocatalysts, such as metal oxides, doped oxides, heterojunctions, and protective layers to enhance charge transfer at both interfaces and in the bulk, thereby lowering the activation energy barriers [7]. In this sense, bismuth vanadate (BiVO_4_) has been pointed out as a promising material, as it presents high chemical stability and a wide absorption range of solar radiation [8–10]. To overcome the limitations associated with the low electrical conductivity of BiVO_4_, sluggish surface kinetics and the recombination of electron–hole pairs [11], it is possible to combine BiVO_4_ with other materials, such as carbon nitrides materials [12].

Carbon nitrides (CNs) can be classified into two main groups: polymeric carbon nitride (PCN) and crystalline carbon nitrides – poly(heptazine imide) (PHI) and poly(triazine imide) (PTI). While both share a fundamental heptazine or triazine-based backbone, they significantly differ in structure, crystallinity, and properties. PCN is the commonly synthesized form of CN due to its simple preparation methods. However, it suffers from low crystallinity, structural disorder, and poor reproducibility, which limit its performance in various applications [13–14]. In contrast, crystalline carbon nitrides, namely PHI and PTI, exhibit superior molecular organization, greater stability, and enhanced functional properties. PHI is composed of heptazine units linked through nitrogen bridges, resulting in a pseudohexagonal 2D lattice. Meanwhile, PTI consists of triazine units, forming a rigid and highly stable framework. This structural arrangement significantly improves charge mobility and light absorption, both of which are critical for catalytic activity [15]. Additionally, PHI and PTI possess an intrinsic ionic nature, unlike the neutral PCN, which enhances their interactions with various substrates and expands their potential applications. A key factor in achieving superior crystallinity of PHI and PTI is ionothermal synthesis, where the use of salts facilitates a well-ordered molecular arrangement and minimizes structural defects [16]. CN materials have been extensively studied as environmentally friendly semiconductors with remarkable photocatalytic properties and facile synthesis [17–21]. Furthermore, it has been demonstrated that incorporating cations during CN synthesis can lead to different structural modifications, varying degrees of crystallinity, and consequently, distinct photocatalytic performance [17–21].

Another strategy to bypass the sluggish kinetics of water oxidation involves employing different processes to deliver the electrons necessary for H_2_ production. In this context, the oxidation of small organic molecules has great potential to replace water oxidation in photoelectrochemical cells [22], as these molecules have a considerably lower standard potential, with the possibility to yield value-added products. Indeed, biomass derivatives, such as ethanol and glycerol, are targeted to become a new route for converting solar energy into fuels [23] at both sides of the cells (i.e., while H_2_ is produced at the cathode, carboxylic acids, aldehydes, and ketones can be produced at the anode [24]). It is expected that glycerol photoelectrochemical oxidation at BiVO_4_ occurs by the following pathway: (i) Photon absorption by BiVO_4_, (ii) electron–hole (*e*^-^–*h*^+^) separation, (iii) electron(s) transfer from glycerol molecule to *h*^+^, and (iv) glycerol deprotonation yielding oxidized products as shown in Equation 1. The products usually described in the literature for this process are 1,3-dihydroxyacetone, glyceraldehyde, formic acid, and glyceric acid [25–26].


[1] $${\text{C}}_{3}{\text{H}}_{8}{\text{O}}_{3}+\text{n}h^{+}\to \text{Products}+{\text{nH}}^{+}$$

Hessel et al. compared the photochemical oxidation of methanol, ethylene glycol, and glycerol in CdS [27], BiVO_4_ [25], and BiVO_4_:Zr,Mo/Pt [25] thin films, concluding that regardless of the material, glycerol oxidation depicted higher photocurrents and provided a protective effect against photocorrosion. The photoelectrochemical activity and selectivity of glycerol oxidation on BiVO_4_ are influenced by several factors, including pH [26], electrolyte cation and anion [28], BiVO_4_ surface crystallography orientation [29], and bismuth-rich domain within the film [30].

Herein, in order to understand the effect of distinct types of CN materials on CN/BiVO_4_ heterojunctions, polymeric (PCN), poly(heptazine imide) (PHI) and poly(triazine imide) (PTI) carbon nitrides were synthesized and employed to fabricate CN/BiVO_4_ electrodes. These heterojunctions were applied to photoelectrochemical glycerol oxidation. Furthermore, the effect of Na, K, and Cs alkaline cations in the PHI structures was investigated and the photoactivity for glycerol oxidation was discussed in terms of CN thermal stability.

## Materials and Methods

### Synthesis of carbon nitrides

The PCN synthesis followed the procedure described by Silva et al. [31]. Briefly, the synthesis was carried out in a hydrothermal reactor for the polymerization of melamine in 25 mL of distilled water at 80 °C for 4 h. At the end of the process, the solvent was evaporated, and the resulting solid underwent thermal treatment at 550 °C for 30 min. The procedure described by Chen et al. [15] was adopted for PHI and PTI synthesis, in which melamine (1 g) was mixed with 10 g of alkali metal chlorides (Li, Na, K, or Cs), and the mixture was heated in a muffle furnace under a N_2_ atmosphere. The heating conditions were adjusted based on the alkali metal chloride used: for Na, the system was heated at 600 °C for 4 h, for Li at 600 °C for 8 h, and for both K and Cs at 550 °C for 4 h. After heating, the product was washed with distilled water and stirred at 95 °C for 3 h. The suspension was then centrifuged at 9000 rpm for 5 min, dried in an oven at 60 °C, and stored for later use. While the synthesis containing LiCl results in the PTI structure, the other salts yield PHI [31–32].

### Carbon nitride deposition onto FTO

An amount of 20 mg of each CN was dispersed into 5 mL of poly(ethylene glycol) (PEG-300, Sigma-Aldrich, ACS reagent, 99.99%) for 30 min in an ultrasound bath (Soni-tech ultrasonic cleaner). Subsequently, 60 µL of a freshly sonicated suspension was drop-cast onto a 1 × 1 cm FTO (Sigma-Aldrich, surface resistivity ≈7 Ω/sq) substrate cleaned/pre-treated according to the protocol described by Malviya et al. [31–32]. The system was spun at 2000 rpm for 30 s in a spin coater device (WS-650Hz-23NPPB). Finally, the C_3_N_4_/FTO layer was placed in a drying oven at a temperature of 300 °C for 1 h.

### Synthesis of heterojunctions

Initially, bismuth was electrodeposited from a bismuth(III) nitrate (Sigma-Aldrich, ACS reagent, 99.99%) solution (0.02 mol·L^−1^ in ethylene glycol) by applying a −1.8 V vs Ag/AgCl/KCl_sat_ potential for five cycles (2 s at open circuit between the cycles) until reaching a charge density of −0.04 C·cm^−2^ at each cycle (total charge of −0.2 C·cm^−2^) [33]. The electrodeposition was performed on both unmodified and FTO-modified subtrates with different CN. Subsequently, 60 µL of a vanadium(III) acetylacetonate (Sigma-Aldrich, ACS reagent, 99.99%) solution (0.2 mol·L^−1^ in DMSO) was drop-cast onto the freshly electrodeposited Bi layer, and the material was heated at 10 °C·min^−1^ up to 500 °C and held for 2 h. After completing this step, the films were soaked into NaOH solution (1.0 mol·L^−1^) for 30 min to remove the excess of unreacted vanadium, washed with distilled water, and dried at ambient temperature.

### Characterization of materials

Prior to deposition on FTO substrates, the different types of CN were structurally and morphologically characterized. Thermogravimetric (TGA) analysis (Netzsch, 209 F3) was performed from 25 to 800 °C at a heating rate of 10 °C·min^−1^ under a synthetic air atmosphere. X-ray diffraction (XRD) patterns (XRD, Rigaku Ultima IV 6000) were acquired from 10° to 80° at a rate of 2°·min^−1^ with a step size of 0.02°. Morphological features and elemental composition were determined by field-emission scanning electron microscopy (FEG-SEM; Zeiss Supra35) coupled with energy-dispersive X-ray spectroscopy (EDS), operating at 15 kV. Optical properties were investigated using ultraviolet–visible spectroscopy in the diffuse reflectance mode (Agilent-Cary Series UV–vis–NIR) in the wavelength range from 800 to 300 nm at 600 nm·min^−1^. The optical energy band gap (*E*_g_) values were estimated using the Kubelka–Munk function applied the Tauc equation (Supporting Information File 1, Equation S1) [34].

The photoelectrochemical characterization was carried out using a potentiostat/galvanostat (PGSTAT302N, Autolab, Metrohm). Measurements were performed in a three-electrode cell containing a Na_2_SO_4_ solution (0.5 mol·L^−1^, pH 6.8), BiVO_4_ or CN/BiVO_4_ as working electrode (WE), a platinum sheet as the counter electrode, and a Ag/AgCl/KCl_sat_ electrode as potential reference. The light source was provided by solar simulator (Oriel, LCS-100, AM-1.5G) with 100 mW·cm^−2^ of irradiance illuminating the back side of the WE through a lateral quartz window in the electrochemical cell (Supporting Information File 1, Figure S1). Linear sweep voltammetry (LSV) curves were acquired in the dark and under simulated solar exposure, ranging from −0.6 to 1.4 V vs Ag/AgCl/KCl_sat_ at 50 mV·s^−1^.

## Results and Discussion

### Physical characterization

**Figure 1:**
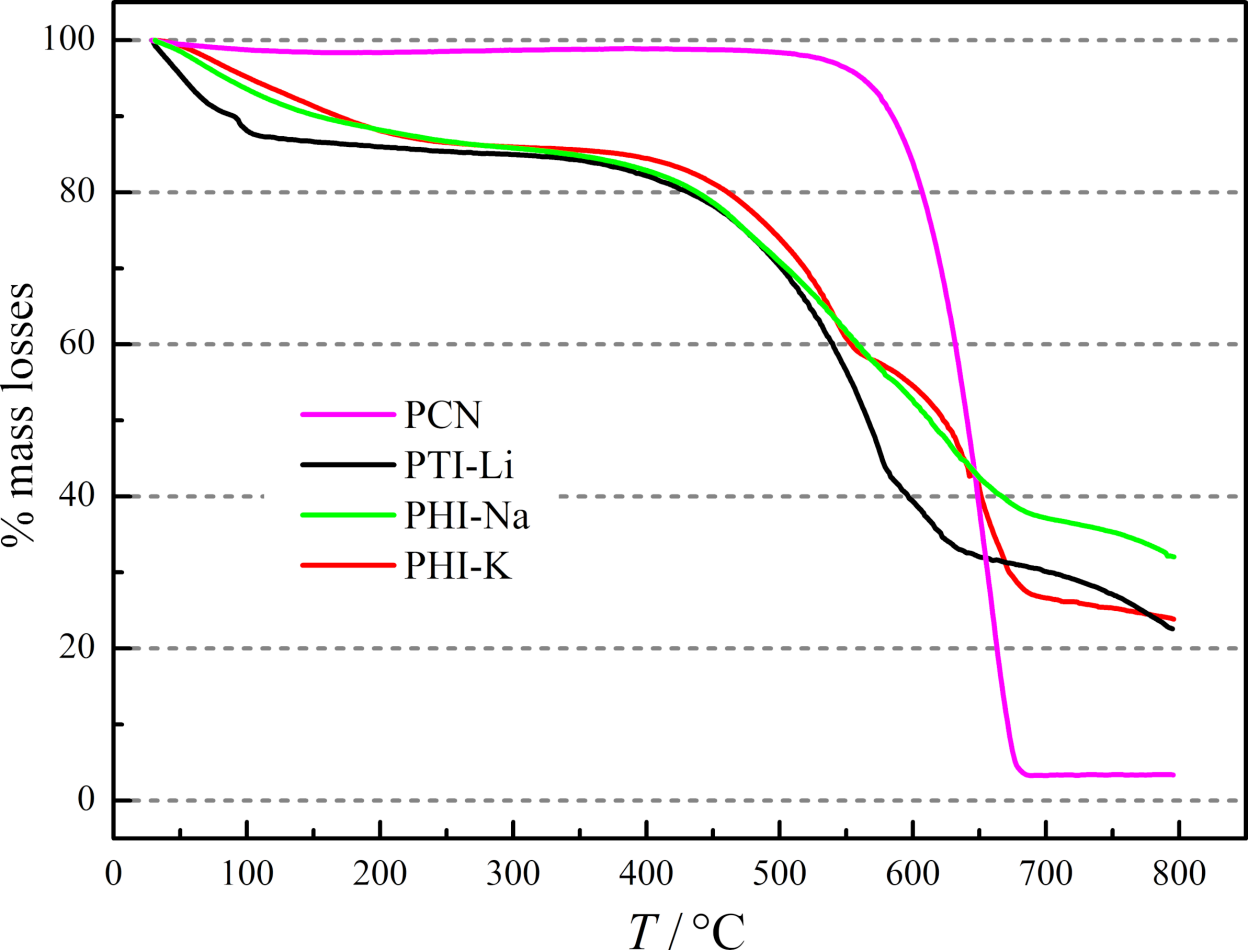
Thermogravimetric analysis of PCN, Li-PTI, Na-PHI, and K-PHI in synthetic air atmosphere.

The CNs thermostability was evaluated by TGA, as shown in Figure 1. PCN presented greater thermal stability at temperatures below 500 °C, the mass losses being more pronounced for temperatures higher than 550 °C, resulting in a residual mass of 3.3% at 700 °C. On the other hand, remaining materials (PTI-Li, PHI-Na, and PHI-K) exhibited mass losses at lower temperatures, which can be divided into two stages: (i) mass losses at *T* < 300 °C are probably due to water in the CN structures, which is connected to the residual water after the last part of the synthesis protocol which involves dissolution in water; (ii) mass losses at *T* > 300 °C are likely connected with the CN degradation, which happens at considerably lower temperatures than those observed for PCN. Interestingly, even at *T* = 800 °C in an oxidizing atmosphere, the materials still maintain 20–35% of their initial mass.

To shed some light on CN structure degradation and the residual nature of the materials at high temperature, a thermal treatment was performed at 500 °C for 2 h and the X-ray diffractograms were collected for the materials with the highest (PCN) and the lowest (PTI-Li) thermal stability. Supporting Information File 1, Figure S2 shows that PCN maintains the XRD pattern after the thermal treatment. On the other hand, PTI-Li suffers structural transformations during thermal treatment, resulting in a strong inhibition of the peaks at 11.8°, 21.2°, and 27.0° and the appearance of an intense and sharp peak at 31.8°. Supporting Information File 1, Figure S3 compares the PTI-Li XRD pattern with simulated XRD patterns of several lithium (hydro)oxides but it is not possible to address the observed features for only one species. Therefore, the material obtained after the thermal treatment seems to be a mixture of unreacted PCN with possibly lithium (hydro)oxides.

**Figure 2:**
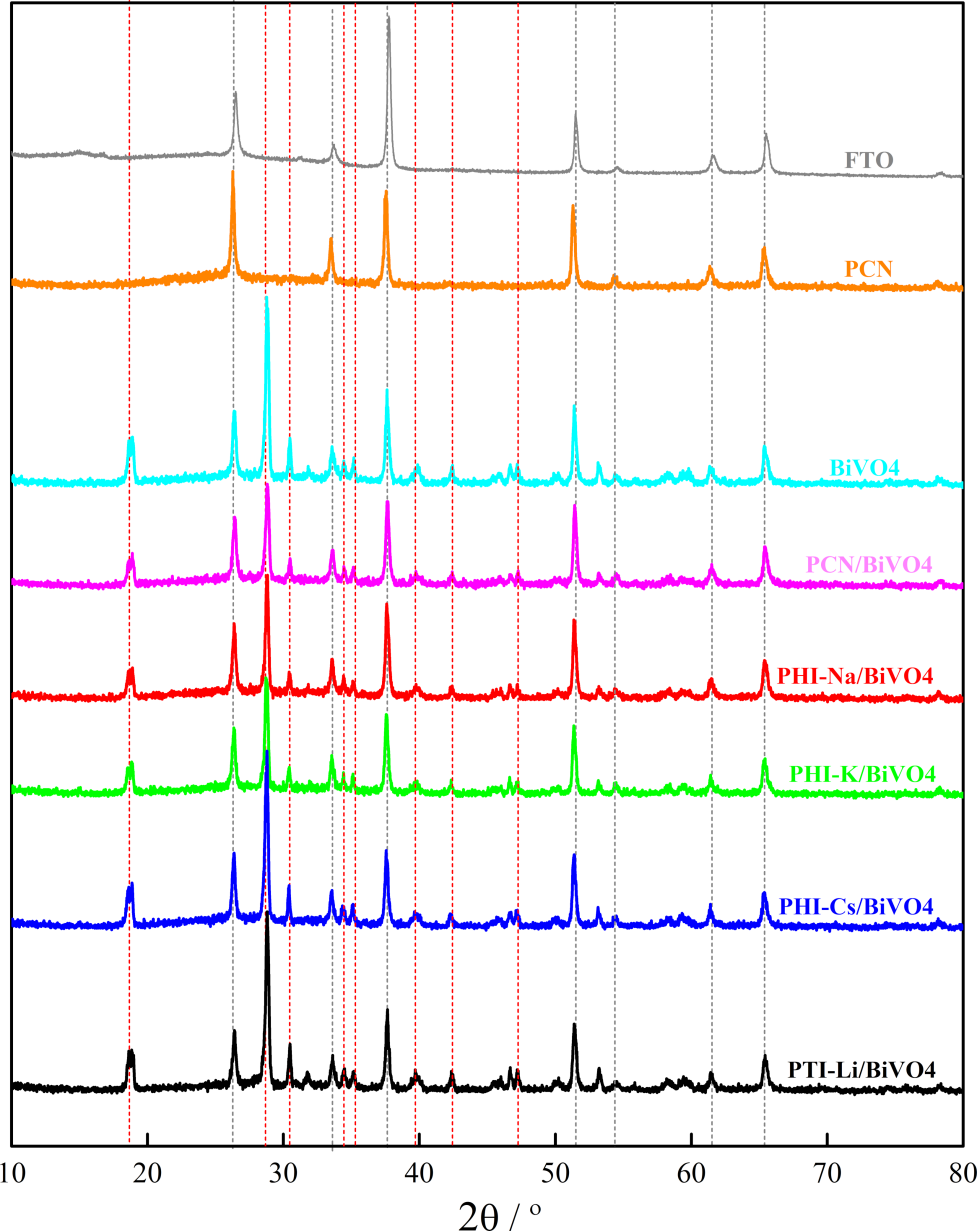
X-ray diffractograms for the material deposited onto FTO substrates.

Figure 2 shows the XRD for the synthesized CNs/BiVO_4_ films and for BiVO_4_ and PCN films onto FTO substrate. The XRD pattern for clean FTO was also included (gray line) making it easier to observe its contribution to the other diffractograms. Notably, in all films, it is possible to observe the diffraction peaks related to the FTO (since the incident beam interacts with all layers of the material). The main FTO contributions are highlighted by the gray dashed lines. The film containing only BiVO_4_ presents a diffraction pattern consistent with the monoclinic structure, according to ICDD file 00-044-0081. The following planes stand out, attested by the peaks that appear at 2θ = 18.7° (011), 28.7° (−121), 30.5° (004), 34.5° (200), 35.2° (202), 39.7° (211), 42.4° (105), and 47.1° (024) in agreement with the assignments reported by Cheng et al. [35]. These contributions are indicated by the red dashed lines. Furthermore, in films containing C_3_N_4_, it is not possible to observe its crystalline phase, likely due to the thin layer of this material on FTO. Supporting Information File 1, Figure S2 brings the CNs XRD for pristine powder forms in which the most intense peaks occur in the range from 26.8 to 28° for PTI-Li, PHI-Na, and PCN. Specifically, for PCN no peaks in this range are observed in Figure 2 for PCN onto FTO. Moreover, BiVO_4_ depicts an intense peak in this region, hindering any possible PCN contribution in PCN/BiVO_4_ XRD. Therefore, for the CNs/BiVO_4_ films, both BiVO_4_ and FTO characteristic diffraction peaks are observed, whereas for the PCN film on FTO only the characteristic FTO diffraction peaks are detected.

**Figure 3:**
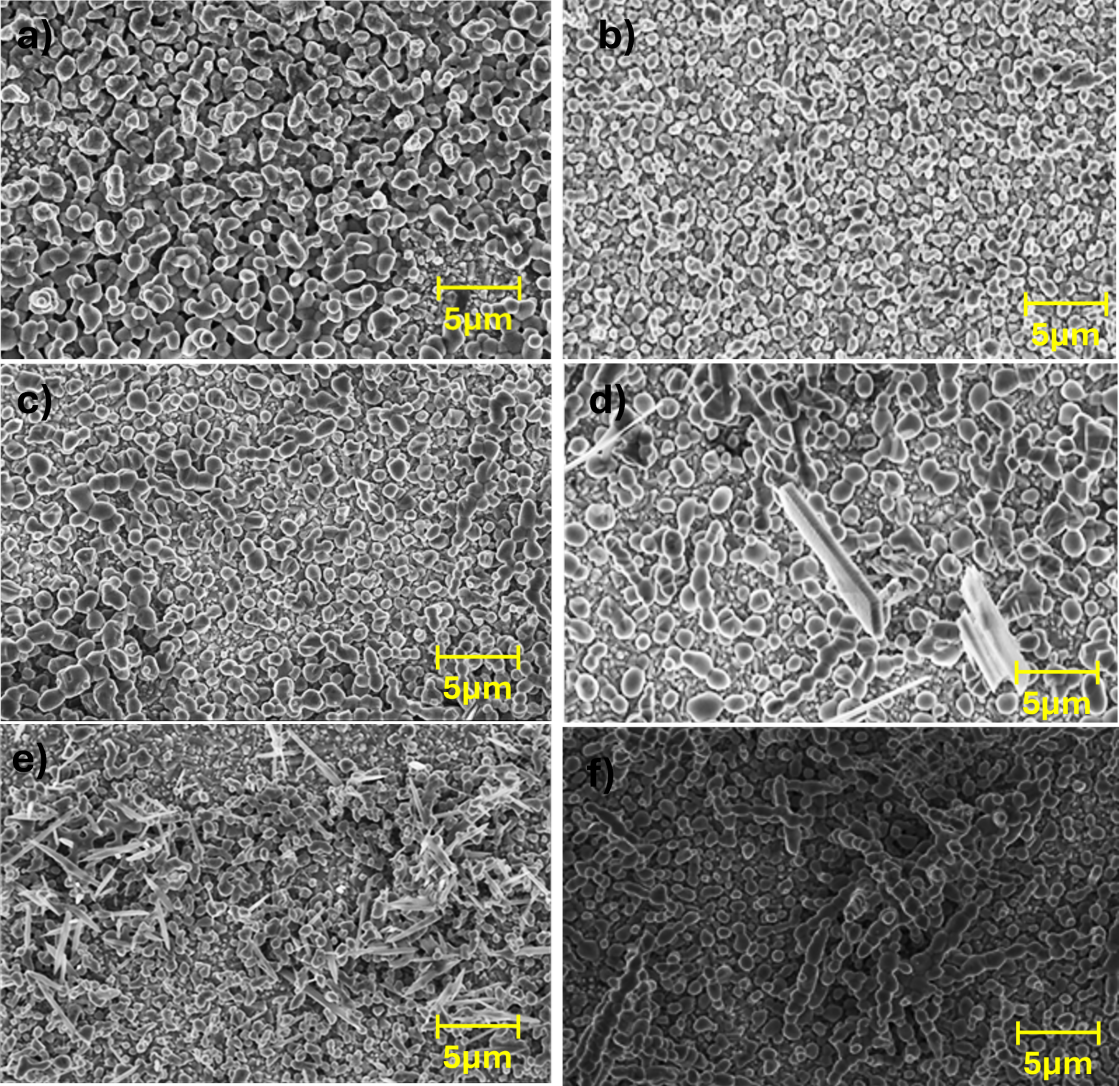
FEG-SEM images for a) BiVO_4_; b) PCN/BiVO_4_; c) PHI-Na/BiVO_4_; d) PTI-Li/BiVO_4_; e) PHI-K/BiVO_4_; and f) PHI-Cs/BiVO_4_ film onto FTO.

FEG-SEM images (Figure 3) were performed on the surface (top view) of the BiVO_4_ and CN/BiVO_4_ films. Globular structures characteristic of bismuth vanadate can be recognized along the surface, these patterns have been reported in the literature for BiVO_4_ synthesized by similar procedures [25,33,36]. For the materials containing heterojunctions, the globular structures occurred in a different way for each material, which could be attributed to nucleation during the heat treatment, depending on the alkali cation present or the CN crystallinity. For instance, in PCN/BiVO_4_ and PHI-Na/BiVO_4_ (Figure 3b and 3c), the globules decreased in size compared to pure BiVO_4_, whereas in PHI-K/BiVO_4_, PHI-Cs/BiVO_4_, and PHI-Li/BiVO_4_, they tended to agglomerate into elongated morphologies. SEM-EDS images for PHI-Li/BiVO_4_ (Supporting Information File 1, Figure S4) revealed that the elongated structures are richer in both vanadium and oxygen than in bismuth. Therefore, it can be concluded these structures are mainly vanadium oxides.

**Figure 4:**
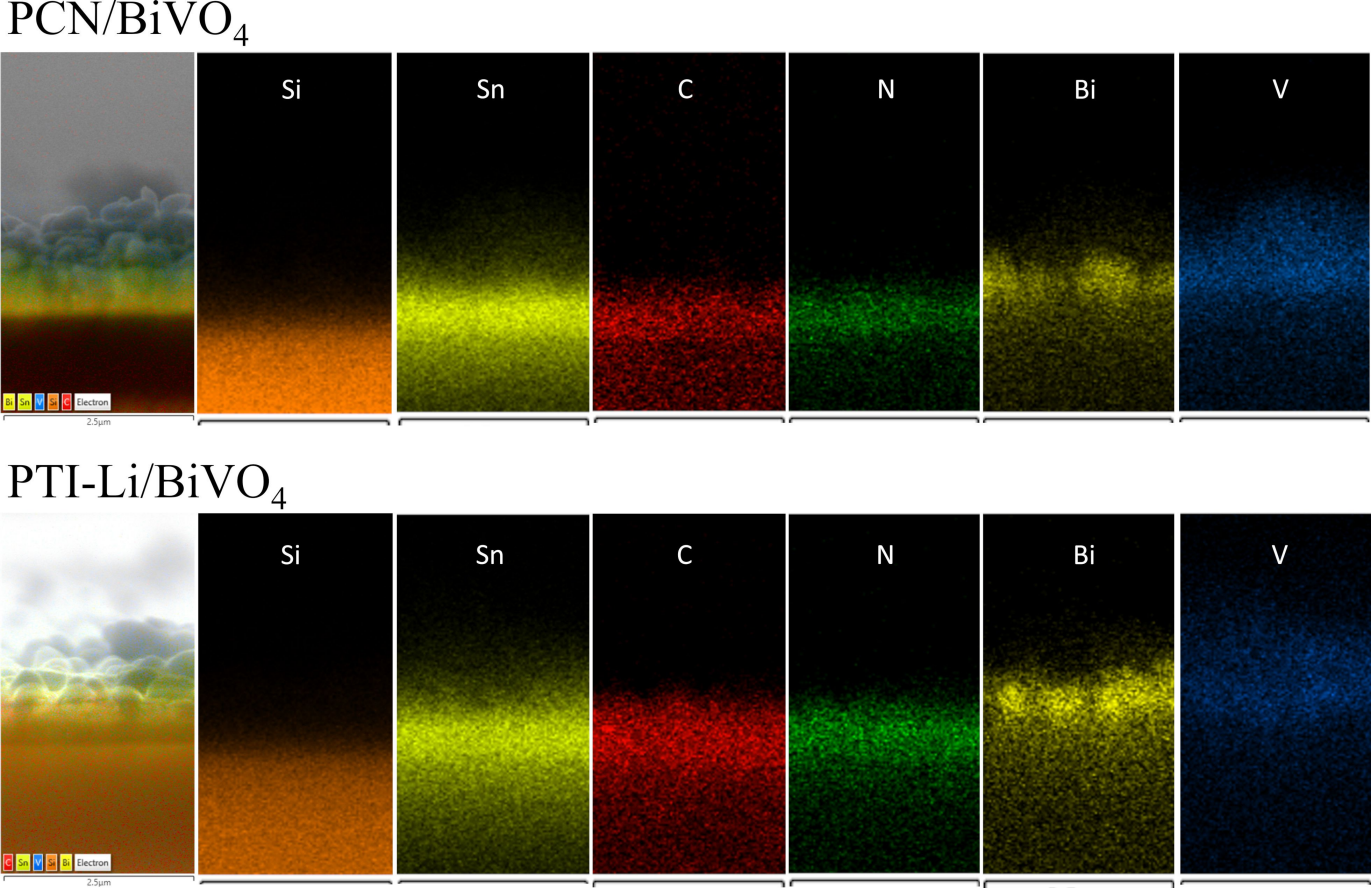
FEG-SEM-EDS images for PCN/BiVO_4_ and PTI-Li/BiVO_4_.

Cross-sectional FEG-SEM-EDS analysis was performed on PCN/BiVO_4_ and PHI-Li/BiVO_4_, (Figure 4) (i.e., the materials that showed the highest and lowest thermal stability, respectively). From the bottom to the top part of both samples, it is possible to recognize the following elements: Si from the glass support, Sn from the FTO layer, both C and N from the CN layer, and both Bi and V from the BiVO_4_ layer. The C and N signals appear to be located in the same region as Sn, which can be interpreted as a thin CN layer distributed among the FTO grains. This is in agreement with the PCN/FTO FEG-SEM analysis (Supporting Information File 1, Figure S5) in which even after the PCN layer deposition, the typical FTO morphology is clearly observed. The Bi and V signals appear above the other elements, confirming that BiVO_4_ is the species in contact with the electrolyte and suggesting the formation of a heterojunction. Finally, the scattered-colored dots in the images indicate that the analysis is near the detection limit of the instrument; therefore, the data should be qualitatively interpreted.

**Figure 5:**
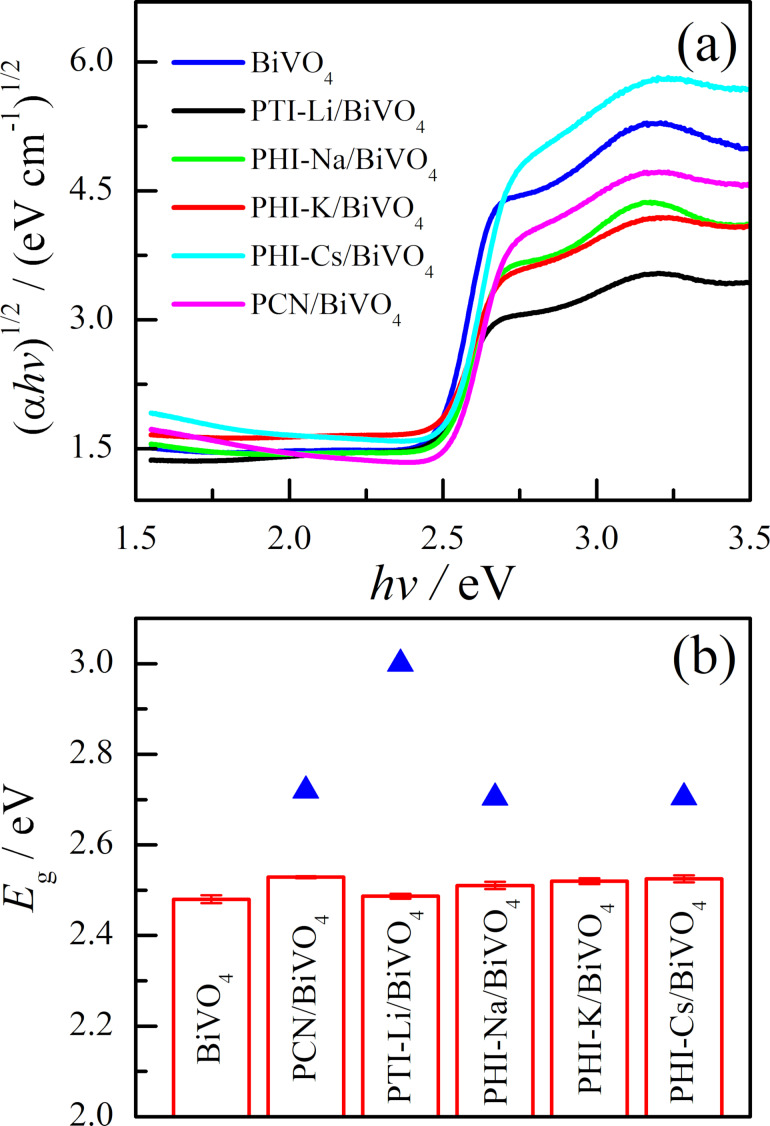
Tauc plot from UV–vis spectra (a) and bandgap energy for both BiVO_4_ and CN/BiVO_4_ films (b). The blue triangles represent the bandgap energy for the CN prior to FTO deposition.

Figure 5 shows the Tauc plots derived from the diffuse reflectance spectra collected for the CN/BiVO_4_ materials, assuming an indirect allowed transition. The bandgap energy values (*E*_g_) were determined by the linear extrapolation of the linear region of the plots to the baseline. The *E*_g_ values were calculated in triplicate for each material, and the mean values, along with their standard deviations, are shown in Figure 5b. Overall, there are no significant variations among the materials, indicating that the underlying CN layer exerts little influence on the *E*_g_ of BiVO_4_, which agrees with previous reports [37–38]. Furthermore, the *E*_g_ values for the CN samples in powder form were also determined. In this case, the *E*_g_ values are higher than those for CN/BiVO_4_ films, as indicated by the blue triangles in Figure 5b, in agreement with the literature values [31].

### Electrochemical characterization

**Figure 6:**
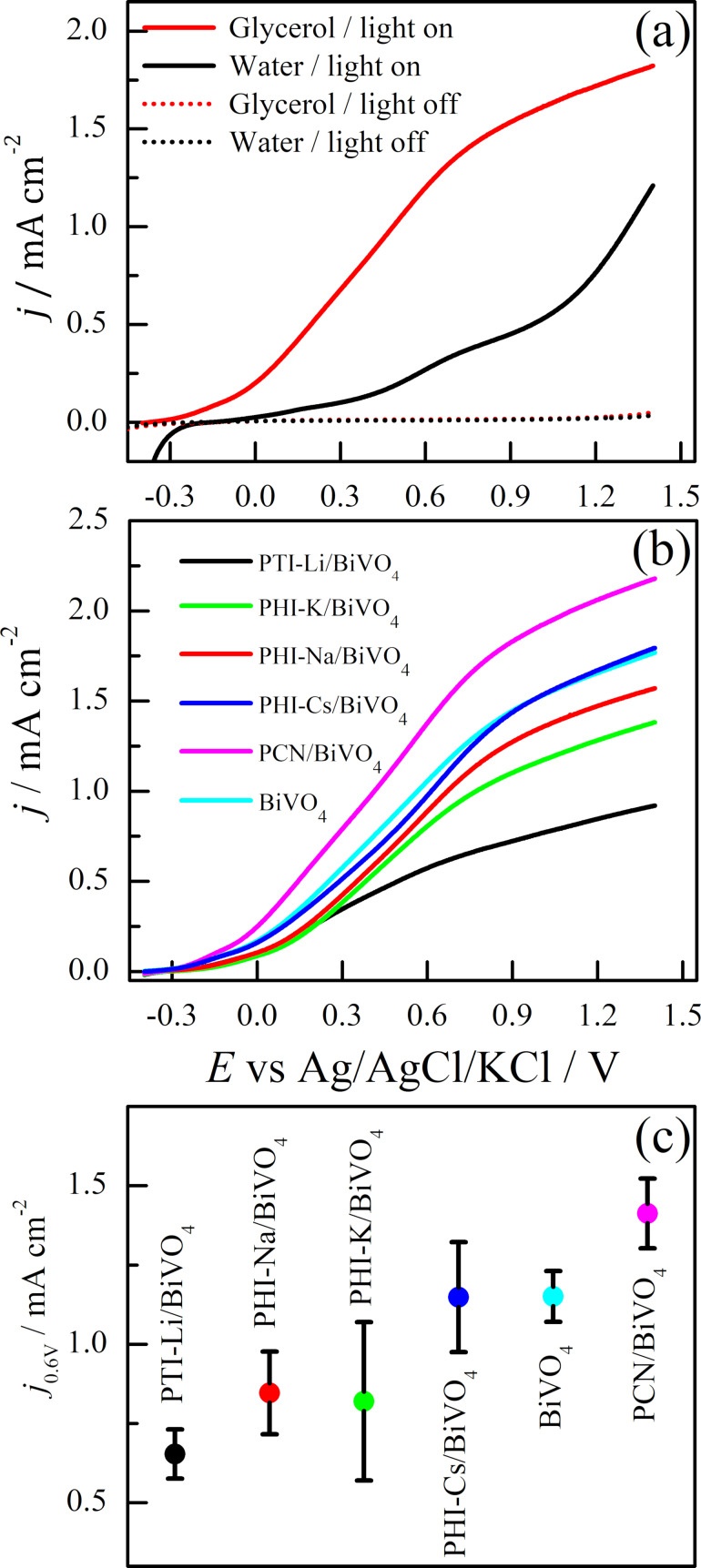
Comparison between photoelectrochemical oxidation of water and glycerol at the BiVO_4_ photoanode under simulated solar illumination (light on) and dark (light off). (a) Glycerol photoelectrochemical oxidation at distinct CN-X/BiVO_4_ films under simulated solar illumination. (b) Photocurrent mean values obtained in (b) at *E* = 0.6 V. (c) The error bars represent the standard deviations from three distinct independently produced films. Other conditions: electrolyte: Na_2_SO_4_ (0.5 mol·L^−1^, pH 6.8) + glycerol (1.0 mol·L^−1^)*v* = 0.05 V·s^−1^_._

Photoelectrochemical activities were evaluated by LSV at a scan rate of 0.05 V·s^−1^ in a Na_2_SO_4_ solution (0.5 mol·L^−1^, pH 6.8). Initially, the activity of the BiVO_4_ film was investigated for water oxidation (in the supporting electrolyte alone) and subsequently for glycerol oxidation after the addition of 1.0 mol·L^−1^ glycerol (Figure 6a). Notably, in the presence of glycerol and simulated light (glycerol/on), the anodic photocurrent onset potential is negatively shifted by 300 mV and the photocurrent densities at 0.6 and 1.5 V are 4.3 and 1.5 times higher than those for water oxidation (water/on), respectively. The dotted lines also confirm that the material is inactive in the dark, regardless of the species in the electrolyte (water/off and glycerol/off).

Intermittent light measurements (chopped light) were also performed to evaluate the influence of pulsed light on the BiVO_4_ photoactivity (Supporting Information File 1, Figures S6 and S7). In the presence of glycerol, the photocurrent closely follows the light on/off transitions, showing stable and sustained values under illumination. In contrast, under water oxidation conditions (i.e., in the absence of glycerol), a sharp current peak appears within fractions of a second upon illumination. These transient current peaks are related to the accumulation of holes (*h*^+^) at the surface of the semiconductor, caused by the sluggish water oxidation kinetics [34].

Following the same protocol employed to test the BiVO_4_, the photoelectrochemical activity of the CN/BiVO_4_ films toward glycerol photoelectrochemical oxidation was also evaluated, and the results are displayed in Figure 6b. For comparison, the curve for the pristine BiVO_4_ film was also included. Interestingly, the onset potential for glycerol oxidation does not depend on the CN type used on the heterojunction, suggesting no doping effect on BiVO_4_. On the other hand, at higher applied potentials, the photocurrent density strongly depends on the CN type. The materials PTI-Li, PHI-Na, and PHI-K combined with BiVO_4_ tend to decrease the overall activity for glycerol oxidation. In turn, PHI-Cs/BiVO_4_ exhibits an activity similar to that of bare BiVO_4_, while PCN/BiVO_4_ displays a higher activity. These photoelectrochemical tests were performed in triplicate using independently prepared electrodes, and the mean photocurrent densities at 0.6 V are shown in Figure 6b. Photocurrent densities under chopped illumination are also presented in Supporting Information File 1, Figures S8–S12 and follow the same trend observed under continuous irradiance. The PCN activity for glycerol was also tested (Supporting Information File 1, Figure S8b) in the same experimental conditions and released photocurrent of ca. 1 μA·cm^−2^. Therefore, the positive effects observed on composite materials come from the synergy between BiVO_4_ and CN.

**Figure 7:**
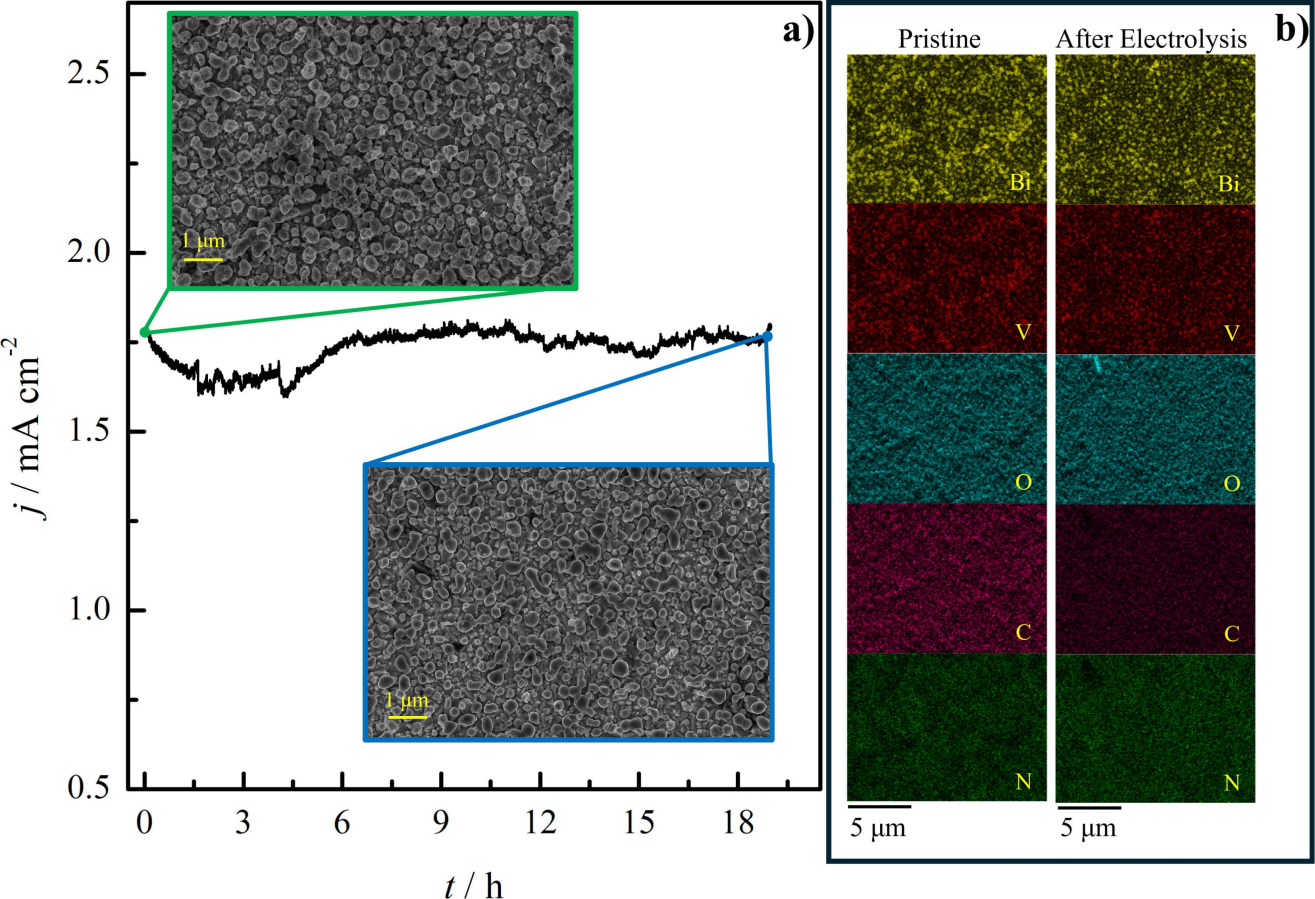
a) Current change at 0.6 V vs Ag/AgCl/KCl for 20 h under simulated solar illumination for PCN/BiVO_4_ in Na_2_SO_4_ + glycerol. The inserts show the FEG-SEM images for pristine (*t* = 0) and post-electrolysis (20 h) electrode surface. b) Elemental analysis (FEG-SEM-EDS mapping) for the pristine and after-electrolysis electrode.

Long-term electrolysis experiments were performed for PCN/BiVO_4_ films at *E* = 0.6 V vs Ag/AgCl/KCl (1.23 V vs RHE) for 20 h under simulated solar illumination (Figure 7a). The photocurrent changes suggest that no corrosion effect or deactivation processes are occurring at the surface during glycerol oxidation. The inserts show FEG-SEM images of the surface before and after electrolysis, confirming that glycerol photoelectrochemical oxidation does not modify the surface condition. Finally, the Figure 7b brings the FEG-SEM-EDS elemental mapping before and after electrolysis. Comparing the images, all elements in pristine material are present after electrolysis (i.e., it has not leached during electrolysis). Therefore, one can conclude the PCN/BiVO_4_ is an active and stable material for glycerol photoelectrochemical oxidation.

During the BiVO_4_ synthesis, an annealing step at 500 °C is necessary to obtain the monoclinic phase. Given the CNs thermal stabilities, significant structural changes are expected, as indicated by TGA analysis. The materials prepared with PTI-Li, PHI-Na, and PHI-K undergo substantial mass loss during this BiVO_4_ synthesis step. In contrast, when coupled with BiVO_4_, PCN, the most stable material among the CNs studied, exhibited a photocurrent density 22% higher than that of bare BiVO_4_ for the glycerol oxidation at 0.6 V vs Ag/AgCl/KCl_sat_ (1.23 V vs RHE).

However, the inherent CNs thermal stability alone cannot explain the behavior of the entire set of tested materials. For instance, the FEG-SEM-EDS indicates the presence of both C and N under the BiVO_4_ layer in the PCN/BiVO_4_ and PTI-Li/BiVO_4_ films. Therefore, a protective effect exerted by the Bi/BiVO_4_ layer over the CNs could be enhancing the thermal stability of the underlying CNs during the BiVO_4_ synthesis, thereby preventing mass loss. Indeed, thermal treatment of the bare CN films deposited onto FTO at 500 °C without the Bi overlayer leads to the complete thermal degradation of the CN layer. Furthermore, we also confirmed that the PTI-Li powder retains its original bandgap energy (*E*_g_ = 3.0 eV) after the thermal treatment at 500 °C for 2 h (Supporting Information File 1, Figure S13).

The films composed of PHI-K/BiVO_4_ and PHI-Cs/BiVO_4_ exhibit activities similar to pristine BiVO_4,_ but the thermal stability of PHI-K/BiVO_4_ is comparable to that of PTI-Li. The literature suggests that the crystallinity of PHI structures increases as the cation radius decreases. This means that PHI synthesized in the presence of smaller cations, such as Na^+^, exhibits a higher degree of crystallinity compared to those prepared with larger cations, such as K^+^. This trend can be attributed to the stronger electrostatic interactions and more efficient packing of smaller cations within the negatively charged imide-linked framework. Smaller cations fit more compactly within the pores and interlayer spaces, promoting a more ordered and rigid structural arrangement. In contrast, larger cations, such as K^+^, create greater spatial distortions, leading to reduced crystallinity and a less well-defined long-range order [15–16,39]. Although it was not possible to confirm these CN structural features by XRD analysis, the photoactivity toward glycerol oxidation follows this trend. In this scenario, the dependence of photoactivity with the CN type could arise from the charge transfer dynamics within the CN layer or at the interface between CN and BiVO_4_, both of which can be tuned by the CN crystalline structure.

Considering that the *E*_g_ values observed for pristine CNs are maintained after thermal treatment under the Bi layer, the low photoactivity for glycerol oxidation at the PTI-Li/BiVO_4_ film could be explained by the high *E*_g_ of PTI-Li, which likely hinders proper band alignment with BiVO_4_. Both PHI-Na and PHI-Cs exhibited similar *E*_g_ values to that of PCN; however, the higher photoactivity of PCN/BiVO_4_ likely arises from its superior thermal stability or more favorable band position. Furthermore, it is possible that the CNs thermally treated under Bi/BiVO_4_ layer exhibit distinct features from those observed for the pristine materials. Unfortunately, techniques such as XRD and Mott–Schottky measurements, typically employed to estimate the crystalline phase and flat band potential, respectively, are challenging to perform on these CN layers with the features of those thermally treated under the Bi layer.

Interestingly, the higher activity observed for glycerol oxidation in PCN/BiVO_4_ is consistent with Gonzaga et al. [12], who reported enhanced BiVO_4_ activity when combined with PCN for textile wastewater detoxification. Thus, coupling BiVO_4_ with PCN appears to be an effective strategy to boost the photocurrent in BiVO_4_-based systems for the oxidation of organic molecules.

## Conclusion

CN/BiVO_4_ heterojunctions were successfully synthesized and characterized using UV–vis spectroscopy, SEM-EDS, XRD, and TGA. These materials were applied to the photoelectrochemical oxidation of glycerol, with particular attention given to the impact of thermal stability. Compared to bare BiVO_4_, heterojunctions based on PTI-Li, PHI-Na, and PHI-K exhibited reduced photoactivity for glycerol oxidation, while PHI-Cs/BiVO_4_ showed comparable performance. Notably, the PCN/BiVO_4_ heterojunction demonstrated enhanced photoelectrochemical activity relative to that of BiVO_4_ alone.

These results highlight the critical role of the CN component in determining the overall performance of the heterojunction. Beyond intrinsic electronic properties, factors such as the nature of the alkali cations, synthesis method, and post-synthesis thermal treatments significantly influence the structural integrity and interfacial behavior of CN materials. Therefore, the design of efficient CN-based heterojunctions must consider not only the initial properties of the CN but also how these properties evolve under processing conditions relevant to device fabrication and operation.

## Supporting Information

File 1 Additional figures.

## Data Availability

All data that supports the findings of this study is available in the published article and/or the supporting information of this article.
