# Supplementary material for: Glycerol photoelectrochemical oxidation reaction at carbon nitrides/BiVO4 materials
Source: Beilstein J Nanotechnol. 2026 Jun 17;17:806–17. doi: 10.3762/bjnano.17.57 (PMC13284753; doi:10.3762/bjnano.17.57)
Supplement: File 1 — Additional figures. [file Beilstein_J_Nanotechnol-17-806-s001.pdf]

BEILSTEIN JOURNAL OF NANOTECHNOLOGY

Supporting Information

for

Glycerol photoelectrochemical oxidation reaction at carbon nitrides/$BiVO_4$ materials

Charles Garcia da Cunha, Isabelle M. D. Gonzaga, Cristian Hessel, Izadora F. Reis, Ivo F. Teixeira, Lucia H. Mascaro and Elton Sitta

Beilstein J. Nanotechnol. **2026,** *17,* 806–817. doi:10.3762/bjnano.17.57

Additional figures

License and Terms: This is a supporting information file under the terms of the Creative Commons Attribution License (https://creativecommons.org/licenses/by/4.0). Please note that the reuse, redistribution and reproduction in particular requires that the author(s) and source are credited and that individual graphics may be subject to special legal provisions.
The license is subject to the *Beilstein Journal of Nanotechnology* terms and conditions: (https://www.beilstein-journals.org/bjnano/terms)

1 – Bandgap energy estimation

The Tauc equation is given by:

$$(\alpha h\nu)^{1/n} = (h\nu - E_g) \quad \text{(S1)}$$

in which α is the absorption coefficient (proportional to the absorbance), E_g is the optical bandgap energy, $h\nu$ is the energy of the incident photons, and n represents the type of electronic transition. Herein, $n = 1/2$ was used for direct allowed transitions and $n = 2$ for indirect allowed transitions.

2 – Experimental Setup

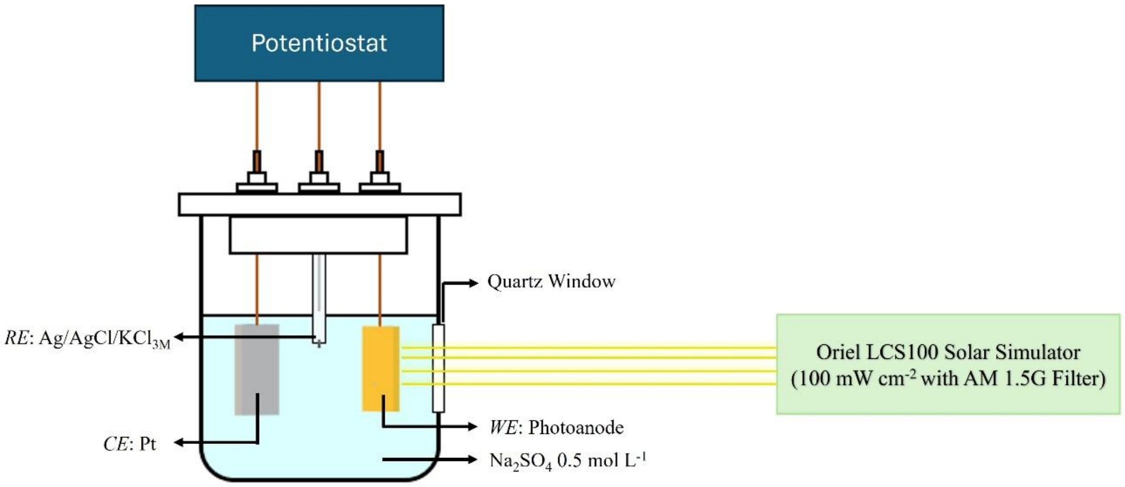

Figure S1: Experimental setup employed for photoelectrochemical experiments.

3 – Physical Characterization

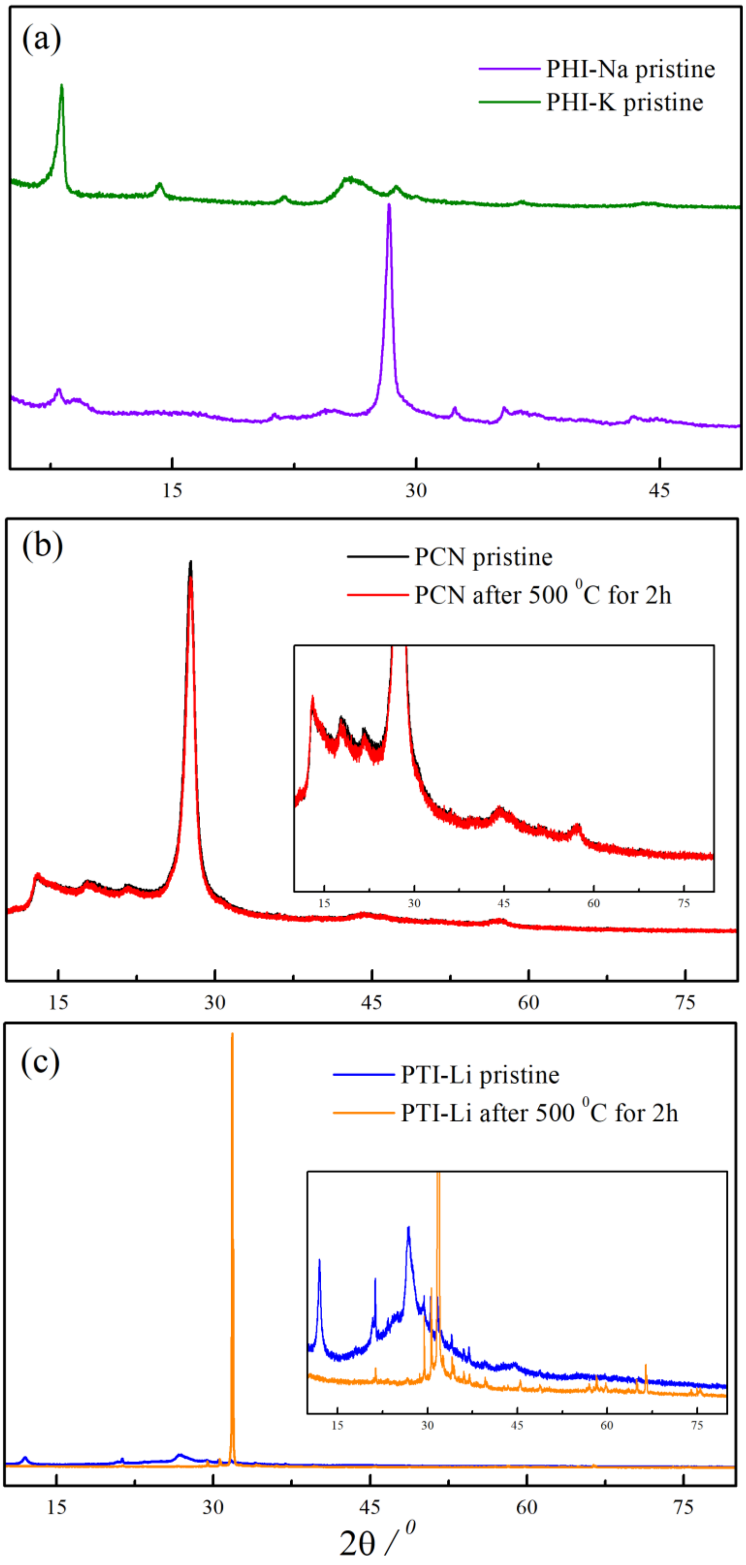

Figure S2: X-ray diffraction patterns for PHI-Na and PHI-K (a), PCN (b), and PTI-Li (c). PCN and PTI-Li XDR were analyzed for both pristine materials and after thermal treatment at 500 °C for 2 h.

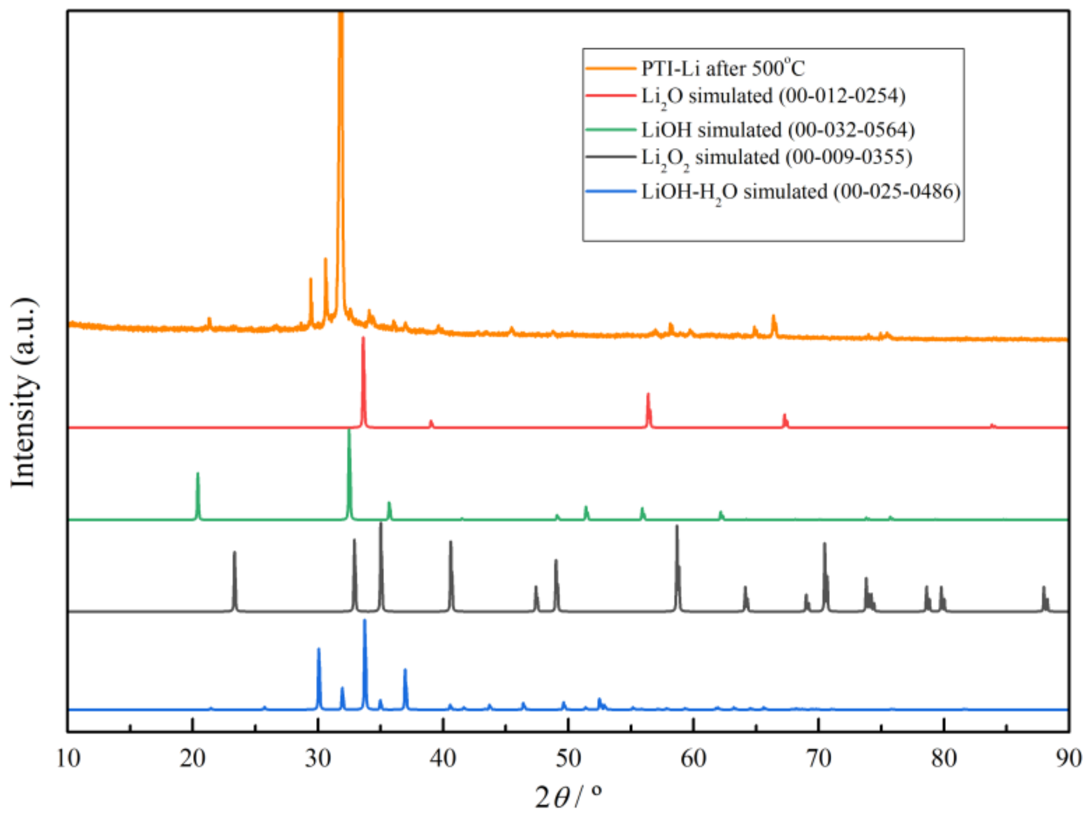

Figure S3: X-ray diffraction patterns for PTI-Li after thermal treatment at 500 °C for 2 h and for some lithium (hydro)oxides.

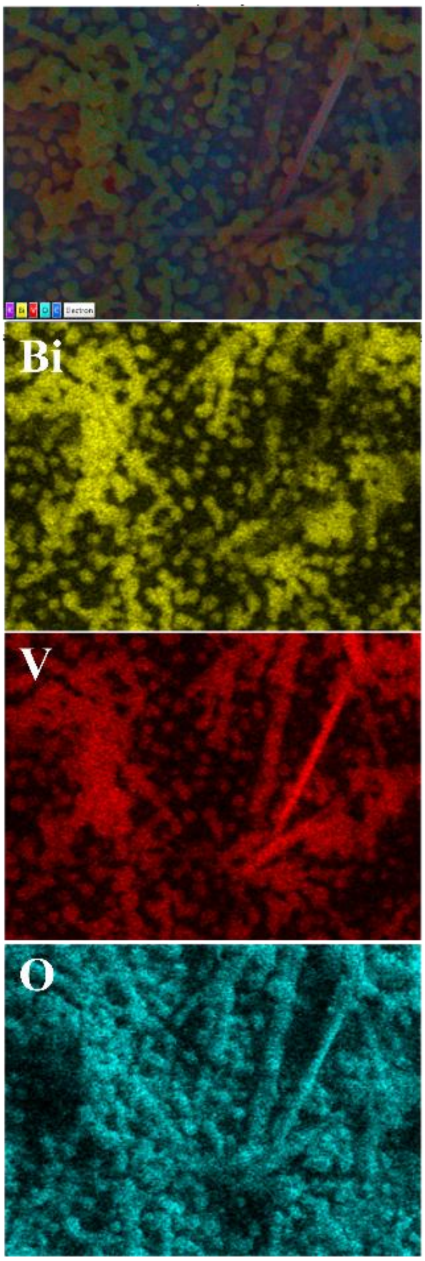

Figure S4: SEM-EDS for PTI-Li/$BiVO_4$.

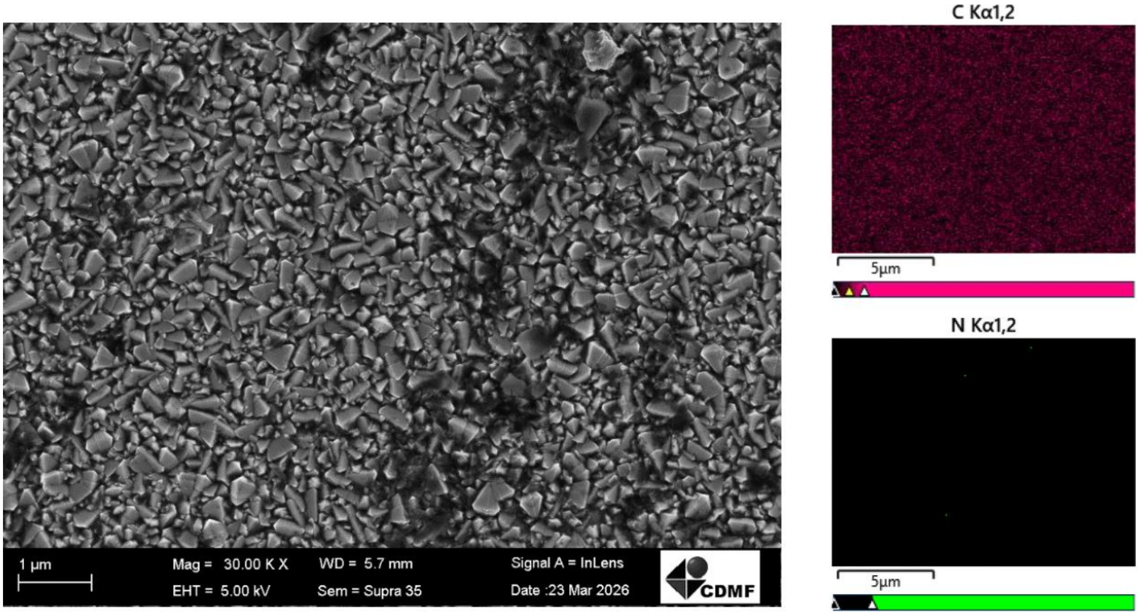

Figure S5: SEM images of PCN onto FTO (left) and carbon and nitrogen elemental mapping obtained by SEM/EDS (right).

4 – Electrochemical Characterization

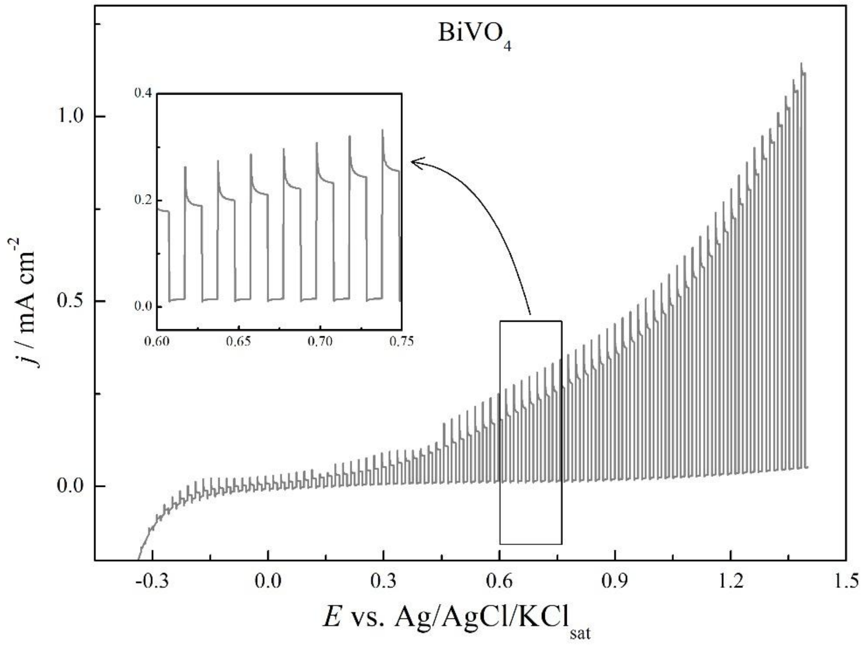

Figure S6: Linear sweep voltammetry (LSV) at 0.05 V·s^{-1} on chopped illumination for the $BiVO_4$ film. Solution condition: Na_2SO_4 (0.5 mol·L^{-1}, pH 6.8).

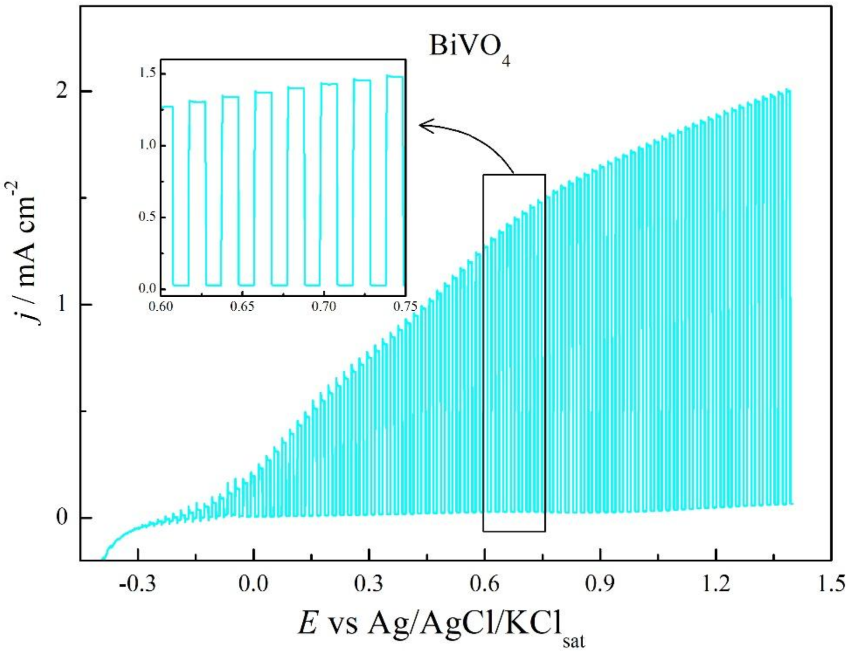

Figure S7: LSV at 0.05 V·s^{-1} on chopped illumination for the $BiVO_4$ film. Solution condition: Na_2SO_4 (0.5 mol·L^{-1}, pH 6.8) + glycerol (1.0 mol·L^{-1}).

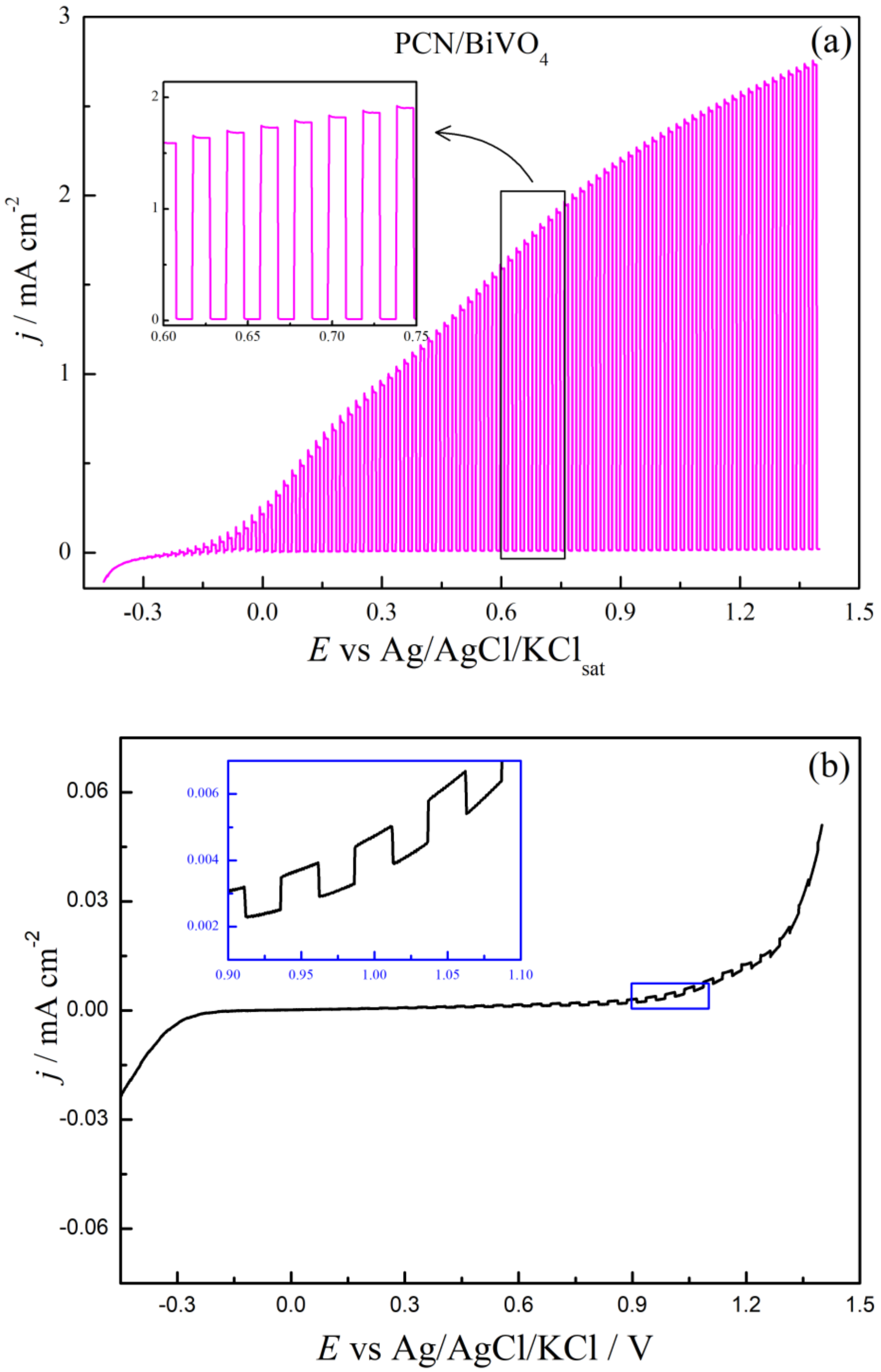

Figure S8: LSV at 0.05 V·s^{-1} on chopped illumination for PCN/$BiVO_4$ (a) and for PCN films (b). Solution condition: Na_2SO_4 (0.5 mol·L^{-1}, pH 6.8) + glycerol (1.0 mol·L^{-1}).

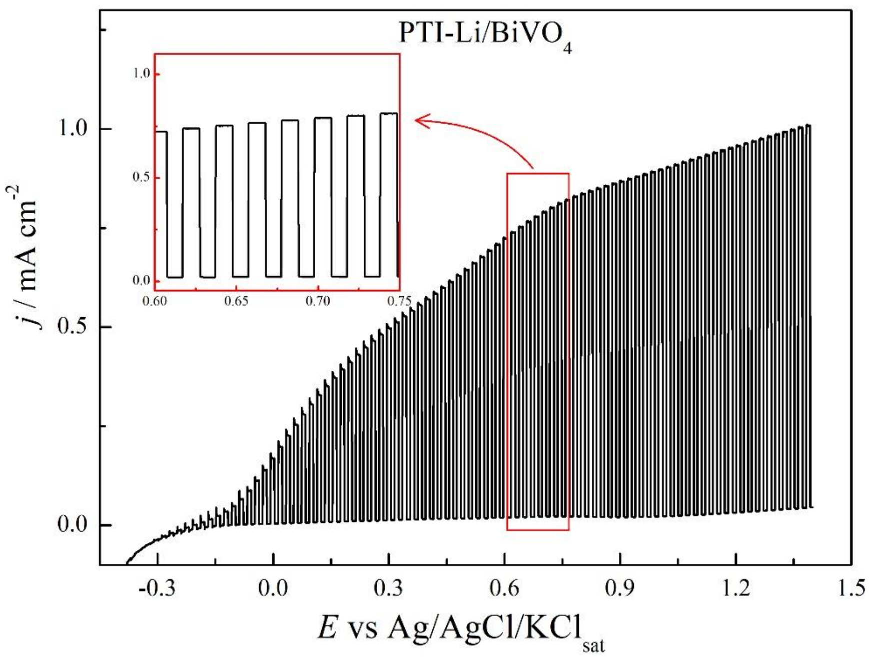

Figure S9: LSV at 0.05 $V{\cdot}s^{-1}$ on chopped illumination for the PTI-Li/$BiVO_4$ film. Solution condition: Na_2SO_4 (0.5 $mol{\cdot}L^{-1}$, pH 6.8) + glycerol (1.0 $mol{\cdot}L^{-1}$).

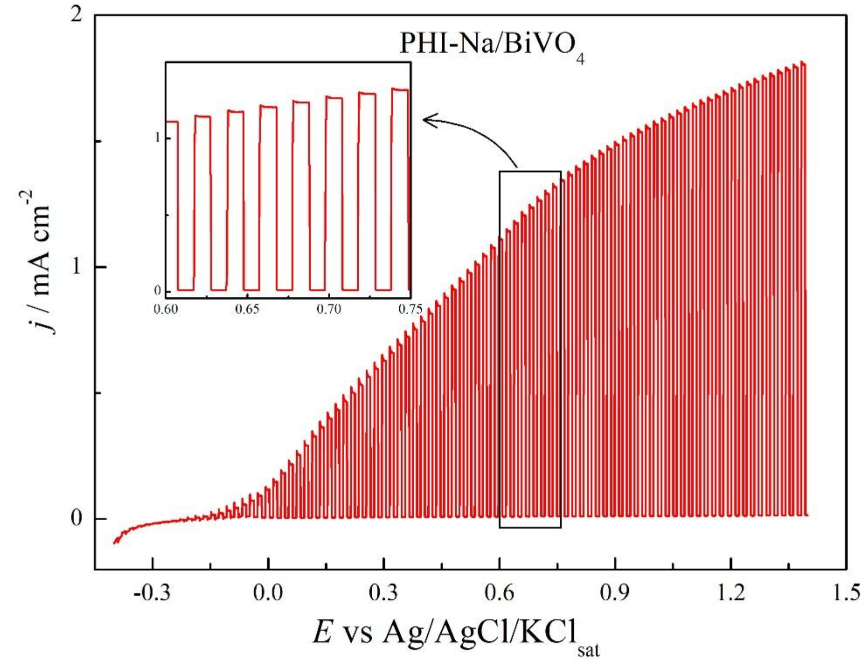

Figure S10: LSV at 0.05 $V{\cdot}s^{-1}$ on chopped illumination for the PHI-Na/$BiVO_4$ film. Solution condition: Na_2SO_4 (0.5 $mol{\cdot}L^{-1}$, pH 6.8) + glycerol (1.0 $mol{\cdot}L^{-1}$).

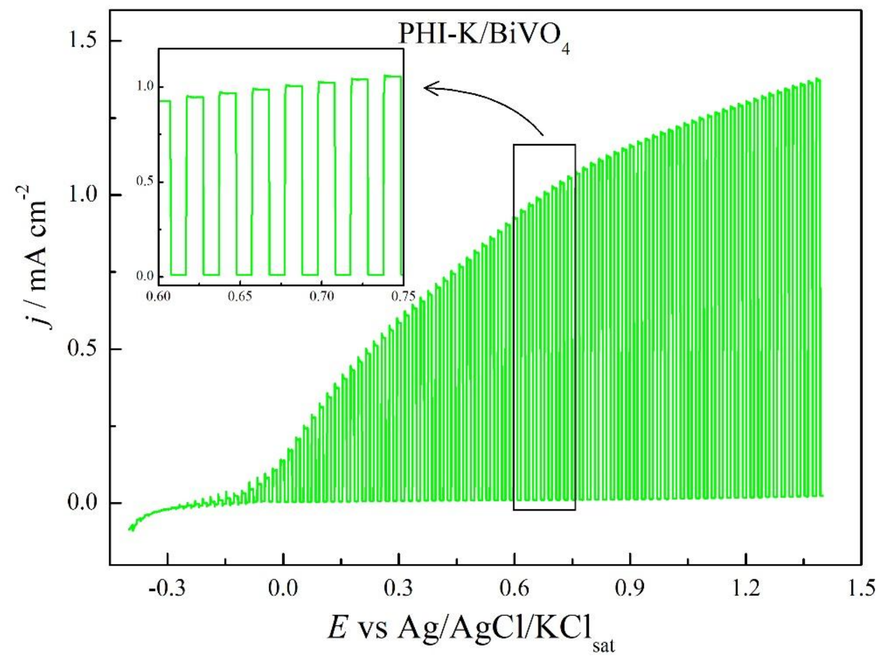

Figure S11: LSV at 0.05 $V \cdot s^{-1}$ on chopped illumination for the PHI-K/$BiVO_4$ film. Solution condition: Na_2SO_4 (0.5 $mol \cdot L^{-1}$, pH 6.8) + glycerol (1.0 $mol \cdot L^{-1}$).

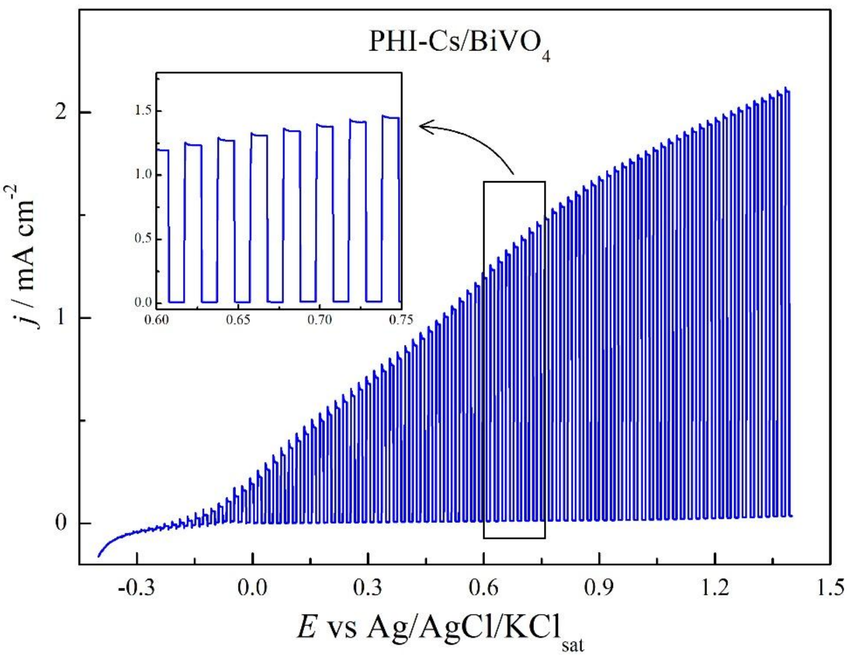

Figure S12: LSV at 0.05 $V \cdot s^{-1}$ on chopped illumination for the PHI-Cs/$BiVO_4$ film. Solution condition: Na_2SO_4 (0.5 $mol \cdot L^{-1}$, pH 6.8) + glycerol (1.0 $mol \cdot L^{-1}$).

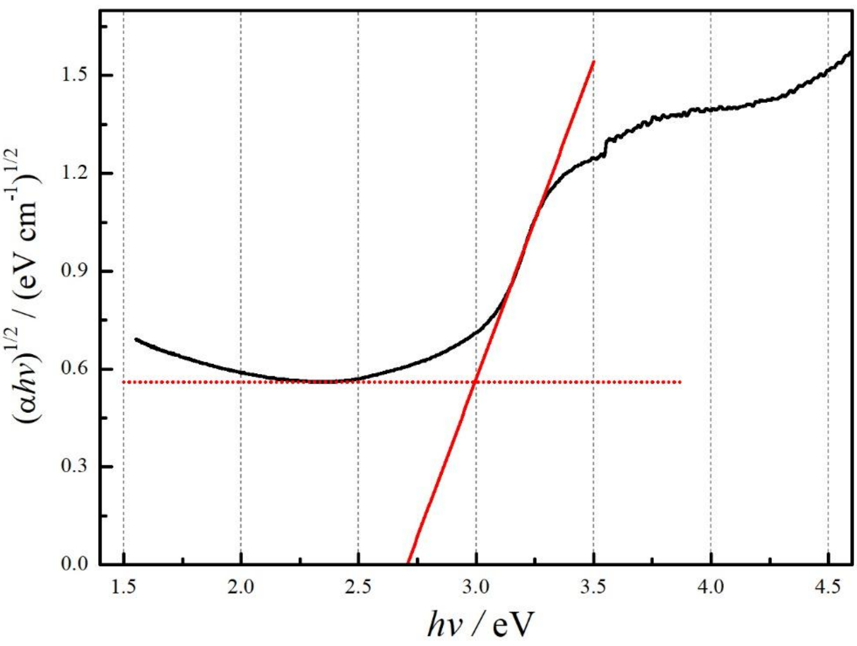

Figure S13: Tauc plot from UV–vis spectrum for PTI-Li after thermal treatment at 500 °C for 2 h.
